# A new mathematical approach to finding global solutions of the magnetic structure determination problem

**DOI:** 10.1038/s41598-018-34443-2

**Published:** 2018-11-01

**Authors:** K. Tomiyasu, R. Oishi-Tomiyasu, M. Matsuda, K. Matsuhira

**Affiliations:** 1 Department of Physics, Tohoku University, Aoba, Sendai, 980-8578 Japan; 2 Faculty of Science, Yamagata University, Yamagata, 990-8560 Japan; 3 JST, PRESTO, Saitama, 332-0012 Japan; 4 Neutron Scattering Division, Oak Ridge National Laboratory, Oak Ridge, TN 37831 USA; 5 Faculty of Engineering, Kyusyu Institute of Technology, Kitakyusyu, 804-8550 Japan

## Abstract

Determination of magnetic structure is an important analytical procedure utilized in various fields ranging from fundamental condensed-matter physics and chemistry to advanced manufacturing. It is typically performed using a neutron diffraction technique; however, finding global solutions of the magnetic structure optimization problem represents a significant challenge. Generally, it is not possible to mathematically prove that the obtained magnetic structure is a truly global solution and that no solution exists when no acceptable structure is found. In this study, the global optimization technique called semidefinite relaxation of quadratic optimization, which has attracted much interest in the field of applied mathematics, is proposed to use as a new analytical method for the determination of magnetic structure, followed by the application of polarized neutron diffraction data. This mathematical approach allows avoiding spurious local solutions, decreasing the amount of time required to find a tentative solution and finding multiple solutions when they exist.

## Introduction

Development of method for determining crystal and magnetic structure is an important issue^1–3^. The magnetic structure of a compound is directly related to the microscopic origins of various intriguing magnetic phenomena observed in physics, chemistry, biology and geology^4–11^. Accurate information on the experimentally determined magnetic structures can be potentially used to effectively design functional materials and opens multiple opportunities for utilizing advanced first-principle calculations and informatics approaches. Furthermore, the responses of the analysed magnetic structure to external factors such as temperature, pressure, electromagnetic field, light and environment are usually related to the corresponding changes in the macroscopic properties of minerals and biomolecules.

**Figure 1:**
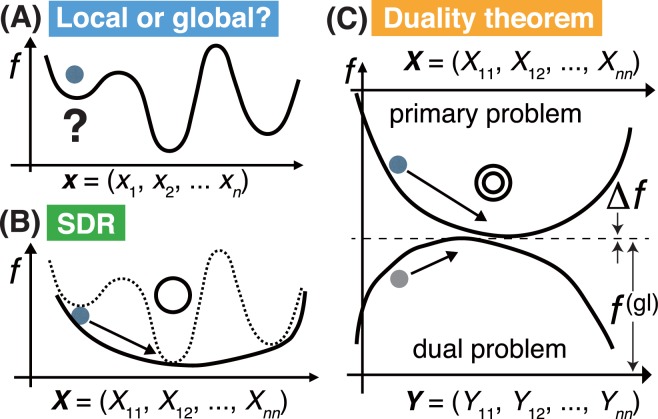
Schematic diagrams illustrating the non-linear optimization process. (**A**) Before relaxation. (**B**) SDR technique. (**C**) Duality theorem combined with SDR. The objective function *f* is globally minimized to the global optimum value ${f}^{({\rm{gl}})}$. When the duality gap ${\rm{\Delta }}f$ is close to zero (corresponding to the square root of the machine epsilon), the global optimization process is complete according to the duality theorem. In panels (**B**) and (**C**), the solid balls represent the paths toward feasible solutions chosen by the interior point method.

Since the pioneering work of Shull *et al*. published in 1951^1^, many studies on the determination of the magnetic structures of various materials by neutron diffraction have been performed. Thus, Izymov, Kovalev and Bertaut developed the irreducible representation (IR) theory, which successfully classified magnetic structures by their magnetic point symmetries obtained through the analysis of neutron diffraction data^12–14^. Moreover, the modern neutron diffractometers exhibit relatively high statistical accuracy and resolution^15,16^. However, analysing neutron diffraction data to determine the correct magnetic structure represents a challenging task. The major issue here is the existence of so-called local solutions to the optimization problem (Fig. 1A). The analysis procedure is mathematically categorized as the nonlinear optimization, in which the obtained diffraction data points are fitted with the function of neutron scattering cross-section characterized by high-dimensional nonlinear parameters such as magnetic moment vectors^17^. After performing nonlinear optimization, it is very difficult to prove that the best solution obtained via various numerical models is the global one. Furthermore, multiple or many solutions are hidden in some cases, and no solutions exist in other cases. To mitigate these issues, global optimization techniques including a simulated annealing method, the Monte Carlo method and genetic algorithms can be used^18–23^. However, none of these approaches can determine the global solution(s) with 100% probability.

In this study, a new analytical method for the determination of magnetic structure is proposed, which allows (i) judging whether the obtained solution is a truly global one; (ii) evaluating the uniqueness of the solution obtained for a given set of experimental data; and (iii) completing the global optimization procedure in a very short time (typically, less than several seconds). Furthermore, the applicability of the proposed method is demonstrated using polarized-neutron powder diffraction data.

## Mathematical Formulation for Applications of Semidefinite Relaxation Method

Relaxation techniques have been previously developed in the field of mathematical programming. In particular, semidefinite relaxation (SDR) combined with semidefinite programming (SDP) has found many applications in applied mathematics and engineering (for example, see Chapter 2.2 in ref.^24^). SDR is an efficient technique for solving nonlinear optimization problems, such as quadratic programs (QPs), namely the minimization of multivariate quadratic polynomials under constraints. It should be noted that SDR could provide both fast convergence and a numerical proof of the global optimality of the obtained solutions using the global convergence property and duality theorem of the convex programming methods, as schematically shown in Fig. 1(B,C).

In the field of optical imaging, Candès *et al*. published a pioneering work (called the PhaseLift method), in which a sparse modelling approach was adopted for general phase retrieval^25^. Furthermore, one of the authors (ROT) developed an SDR-based mathematical approach to investigate whether a crystal structure could be uniquely identified using only diffraction data and independent atomic model^26^.

In this study, this method was applied to magnetic structure analysis by replacing the optimized parameters with magnetic moments. The problem of magnetic structure determination can be mathematically described by the following set of *N* quadratic equations:

1 $${I}_{{\rm{mag}},{\rm{obs}}}({Q}_{k})\approx {I}_{{\rm{mag}},{\rm{cal}}}({Q}_{k})={{\boldsymbol{x}}}^{{\rm{T}}}{S}_{k}{\boldsymbol{x}}=\sum _{1\le i,j\le n}{S}_{k}(i,j){x}_{i}{x}_{j}\,(1\,\le \,k\,\le N),$$

where ${I}_{\mathrm{mag},\mathrm{obs}}({Q}_{k})\,$and ${I}_{\mathrm{mag},\mathrm{cal}}({Q}_{k})$ denote the observed and calculated integrated magnetic diffraction intensities at the magnetic reflection ${Q}_{k}={h}_{{\rm{mag}}}\,{k}_{{\rm{mag}}}\,{l}_{{\rm{mag}}}$, respectively; ***x*** = (*x*_1_, …, *x*_*n*_)^T^ and ${S}_{k}$ represents the coefficient matrix numerically obtained using the scattering cross-section formula for each ${Q}_{k}$, which consists of the absolute intensity scale factor, magnetic form factor, Lorentz factor, multiplicity and structural weight factor (the details of this formula are summarized in Supplementary Information).

The symbol $\approx$ indicates that each ${I}_{{\rm{mag}},{\rm{obs}}}({Q}_{k})$ value includes an experimental error. In order to incorporate all errors, the following optimization problem is solved:

2 $$\{\begin{array}{c}{\rm{Minimize}}\sum _{k=1}^{N}\frac{|{I}_{{\rm{mag}},{\rm{obs}}}({Q}_{k})-{{\boldsymbol{x}}}^{{\rm{T}}}S{\boldsymbol{x}}|}{{\rm{Err}}[{I}_{{\rm{mag}},{\rm{obs}}}({Q}_{k})]},\\ {\rm{subject}}\,{\rm{to}}:|{x}_{i}|\le {R}_{i}(1\le i\le n).\end{array}$$

The inequality $|{x}_{i}|\le {R}_{i}$ can be also removed if the permitted range of $|{x}_{i}|$ (${R}_{i}$, for example, the upper limit of magnetic moment) is uncertain while maintaining the validity of the subsequent discussion of the SDR and SDP techniques.

Equation (2) is classified as a so-called *l*^1^-norm minimization problem, which is equivalent to the following form of the quadratic programming problem:

3 $$\{\begin{array}{c}{\rm{Minimize}}\,\sum _{k=1}^{N}\,\frac{{\eta }_{k}^{+}+{\eta }_{k}^{-}}{2}\\ {\rm{subject}}\,{\rm{to}}:\frac{{I}_{{\rm{mag}},{\rm{obs}}}({Q}_{k})-{{\boldsymbol{x}}}^{{\rm{T}}}{S}_{k}{\boldsymbol{x}}}{{\rm{Err}}[{I}_{\mathrm{mag},\mathrm{obs}}({Q}_{k})]}=\frac{{\eta }_{k}^{+}-{\eta }_{k}^{-}}{2}(1\le k\le N),\\ {{x}_{i}}^{2}+{y}_{i}={{R}_{i}}^{2},\,{y}_{k}\ge 0\,(1\le i\le n),\\ {\eta }_{k}^{+}\ge 0,{\eta }_{k}^{-}\ge 0\,(1\le k\le N).\end{array}$$

The problems described by equations (2) and (3) are equivalent since they have the same set of solutions ***x*** and the minimized values of the objective functions (more details are provided in Supplementary Information). The basic strategy utilized in this work is to apply SDR to equation (3) in order to solve equation (2) and then provide a computational proof on the global convergence property of the solution. The least-squares minimization of the $\,\sum _{k=1}^{N}{|({I}_{\mathrm{mag},\mathrm{obs}}({Q}_{k})-{{\boldsymbol{x}}}^{T}S{\boldsymbol{x}})/{\rm{Err}}[{I}_{\mathrm{mag},\mathrm{obs}}({Q}_{k})]|}^{2}$ function is avoided because a small increase in the polynomial degree considerably magnifies the size of the SDR problem (it increases proportionally to the power of *N*). Thus, it is possible to locate the global optimum by performing *l*^1^-norm minimization, which can be subsequently used as the initial parameter of the normal least-squares method to calculate the refined parameters and then compare them with literature data.

## Application to Experimental Data Analysis

### Experimental results

The developed SDR method was verified by applying it to the experimental data obtained for pyrochlore Nd_2_Ir_2_O_7_, which served as a proximate material for a three-dimensional Weyl semimetal and a component of spintronic devices on the basis of its geometrically frustrated magnetism and 5*d*-electron configuration^27–32^. This state theoretically corresponds to the all-in all-out type of magnetic structure described by the magnetic propagation vector ***k***_mag_ = (0, 0, 0), in which all the magnetic moments are oriented either towards the centre of the participating tetrahedron or in the opposite direction^30,32^. This prediction was experimentally confirmed in previous unpolarized neutron diffraction studies^33,34^. However, the superposition of the nuclear and magnetic reflections observed for the magnetic structures with ***k***_mag_ = (0, 0, 0) produces ambiguous results during their separation. Furthermore, it is not possible to mathematically prove with 100% certainty the absence of other acceptable solutions to the problem of magnetic structure determination. Thus, in this work, polarized neutron diffraction studies were performed for this material, and the obtained magnetic structure was verified mathematically.

**Figure 2:**
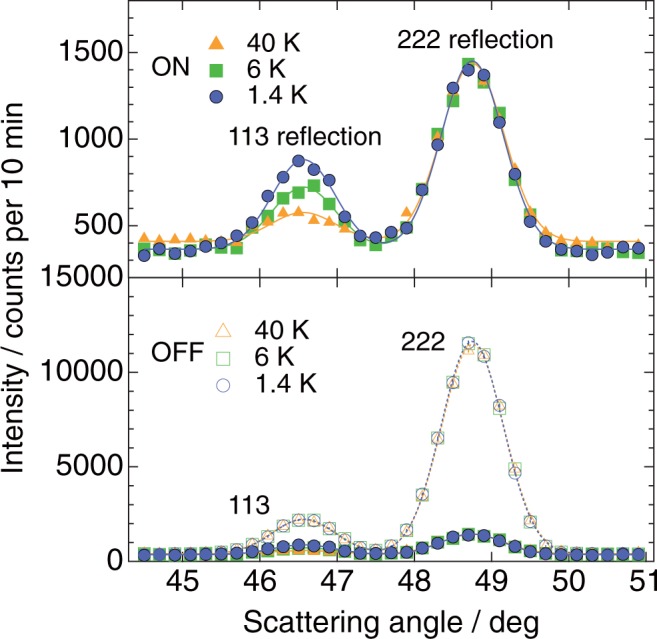
Typical polarized neutron diffraction data obtained for pyrochlore Nd_2_Ir_2_O_7_. ON/OFF denotes the spin-flip/non-spin-flip scattering channel. The lower panel displays both the overall OFF and ON data, whereas the upper panel depicts the low-intensity ON data at a higher magnification. The solid and dotted curves show the results of Gaussian fitting.

Figure 2 shows the representative neutron diffraction data obtained for Nd_2_Ir_2_O_7_ at the minimum temperature *T* = 1.4 K. The non-spin-flip and spin-flip parameters *I*_OFF_ and *I*_ON_ roughly correspond to the nuclear and magnetic reflection components *I*_nuc_ and *I*_mag_, respectively. The magnitude of *I*_OFF_ is systematically larger than *I*_ON_ at all temperatures, thus confirming the necessity of conducting polarized neutron diffraction experiments. For *I*_ON_, the intensity of the 113 reflection increases with decreasing temperature, indicating the existence of strong temperature dependence for this reflection, which is not observed for the 222 reflection. Moreover, as the flipping ratio of the neutron beam in the actual experiments is finite, the magnitudes of *I*_ON_ and *I*_OFF_ can be expressed by the following formulas:

$$\{\begin{array}{c}{I}_{{\rm{OFF}}}=(1-{r}_{{\rm{mix}}}){I}_{{\rm{nuc}}}+\,{r}_{{\rm{mix}}}{I}_{{\rm{mag}}},\,\\ {I}_{{\rm{ON}}}={r}_{{\rm{mix}}}{I}_{{\rm{nuc}}}+\,(1-{r}_{{\rm{mix}}}){I}_{{\rm{mag}}},\,\end{array}$$

where *r*_mix_ = *N*_−_/(*N*_+_ + *N*_−_) is the mixing rate, and *N*_+_ (*N*_−_) denotes the number of majority-spin (minority-spin) neutrons. The value of *r*_mix_ is selected to satisfy the condition *I*_mag_ = 0; hence, *I*_ON_ = *r*_mix_·*I*_nuc_ at the paramagnetic *T* = 40 K. As a result, the pure magnetic intensity *I*_mag_ can be determined by combining *I*_OFF_ and *I*_ON_. A more detailed explanation and data obtained for other reflections are presented in Supplementary Information, while the calculated *I*_mag,obs_ values are listed in Table 1.

**Table 1:** Experimentally observed and calculated integrated magnetic scattering intensities. The symbol ± denotes the experimental errors.

*h*_mag_ *k*_mag_ *l*_mag_	*I*_mag,obs_ (1.4 K)	*I*_mag,cal,Γ3_	*I*_mag,cal,Γ5_	*I*_mag,cal,Γ7_	*I*_mag,cal,Γ9_
111	0 ± 53	0	7	0	457
200	0 ± 36	0	0	0	1
220	932 ± 54	932	932	0	1
113	1796 ± 68	1795	4	0	860
222	0 ± 63	0	0	0	0
400	0 ± 37	0	0	0	0
331	349 ± 54	580	5	0	349
420	884 ± 62	588	541	0	2

### Magnetic structure analysis results

The arbitrary magnetic structure with ***k***_mag_ = (0, 0, 0) in the space group $Fd\bar{3}m$ is described as the function of ***x***_arb_ = (*m*^(Nd)^_1_, *…*, *m*^(Nd)^_12_, *m*^(Ir)^_1_, *…*, *m*^(Ir)^_12_) containing *n*_arb_ = 24 variables generated by the eight magnetic atoms of the unit cell and three Cartesian coordinates. Here, the magnetic symmetry was classified by performing IR analysis (this procedure is generally not required for the proposed method; however, it may be potentially useful because of the reduction of the number of variables if the number of observed reflection points is limited). The possible magnetic structures are described by the representations Γ_Nd_ = Γ_3_ + 2Γ_5_ + 3Γ_7_ + 6Γ_9_ and Γ_Ir_ = Γ_3_ + 2Γ_5_ + 3Γ_7_ + 6Γ_9_, where Γ_3_ corresponds to the all-in all-out type of the magnetic structure, and the coefficients denote the numbers of basis vectors summarized in Table 2^18,35^. Hence, the total numbers of variables are reduced to *n*_Γ3_ = 2, *n*_Γ5_ = 4, *n*_Γ7_ = 6 and *n*_Γ9_ = 12. Furthermore, representation Γ_9_ includes six ferromagnetic basis vectors. However, the Nd_2_Ir_2_O_7_ structure exhibits only extremely weak spontaneous magnetization (around 10^−4^*μ*_B_/formula unit)^29^, which is significantly below the detection limit of the neutron diffraction technique. Therefore, the ferromagnetic basis vectors are precluded and the value of *n*_Γ9_ is further reduced from 12 to 6 during the analysis of the neutron diffraction data.

**Table 2:** Basis vectors of the magnetic structures represented by the Γ_3_, Γ_5_, Γ_7_ and Γ_9_ IRs^18,35^. The symbol – in the Γ_9_ IR indicates that the ferromagnetic basis vectors are precluded.

IRs	Variables *x*	Nd1, Ir1	Nd2, Ir2	Nd3, Ir3	Nd4, Ir4
		(0,0,0), (1/2,0,0)	(1/4,1/4,0), (3/4,1/4,0)	(0,1/4,1/4), (1/2,1/4,1/4)	(1/4,0,1/4), (3/4,0,1/4)
Γ_3_	*m*^(Γ3,Nd)^_1_, *m*^(Γ3,Ir)^_1_	$(1,1,1)/\sqrt{3}$	$(-\,1,-\,1,1)/\sqrt{3}$	$(1,-\,1,-\,1)/\sqrt{3}$	$(-\,1,1,-\,1)/\sqrt{3}$
Γ_5_	*m*^(Γ5,Nd)^_1_, *m*^(Γ5,Ir)^_1_	$(1,-\,1,0)/\sqrt{2}$	$(-\,1,1,0)/\sqrt{2}$	$(1,1,0)/\sqrt{2}$	$(-\,1,-\,1,0)/\sqrt{2}$
	*m*^(Γ5,Nd)^_2_, *m*^(Γ5,Ir)^_2_	$(1,1,-\,2)/\sqrt{6}$	$(-\,1,-\,1,-\,2)/\sqrt{6}$	$(1,-\,1,2)/\sqrt{6}$	$(-\,1,1,2)/\sqrt{6}$
Γ_7_	*m*^(Γ7,Nd)^_1_, *m*^(Γ7,Ir)^_1_	$(0,-\,1,1)/\sqrt{2}$	$(0,-\,1,-\,1)/\sqrt{2}$	$(0,1,-\,1)/\sqrt{2}$	$(0,1,1)/\sqrt{2}$
	*m*^(Γ7,Nd)^_2_, *m*^(Γ7,Ir)^_2_	$(1,\,0,-\,1)/\sqrt{2}$	$(1,0,1)/\sqrt{2}$	$(-\,1,0,-\,1)/\sqrt{2}$	$(-\,1,0,1)/\sqrt{2}$
	*m*^(Γ7,Nd)^_3_, *m*^(Γ7,Ir)^_3_	$(-\,1,1,0)/\sqrt{2}$	$(1,-\,1,0)/\sqrt{2}$	$(1,1,0)/\sqrt{2}$	$(-\,1,-\,1,0)/\sqrt{2}$
Γ_9_	*m*^(Γ9,Nd)^_1_, *m*^(Γ9,Ir)^_1_	$(1,1,0)/\sqrt{2}$	$(-\,1,-\,1,0)/\sqrt{2}$	$(-\,1,1,0)/\sqrt{2}$	$(1,-\,1,0)/\sqrt{2}$
	*m*^(Γ9,Nd)^_2_, *m*^(Γ9,Ir)^_2_	$(0,1,1)/\sqrt{2}$	$(0,1,-\,1)/\sqrt{2}$	$(0,-\,1,-\,1)/\sqrt{2}$	$(0,-\,1,1)/\sqrt{2}$
	*m*^(Γ9,Nd)^_3_, *m*^(Γ9,Ir)^_3_	$(1,0,1)/\sqrt{2}$	$(1,0,-\,1)/\sqrt{2}$	$(-\,1,0,1)/\sqrt{2}$	$(-\,1,0,-\,1)/\sqrt{2}$
	—	$(1,0,0)$	$(1,0,0)$	$(1,0,0)$	$(1,0,0)$
	—	$(0,1,0)$	$(0,1,0)$	$(0,1,0)$	$(0,1,0)$
	—	$(0,0,1)$	$(0,0,1)$	$(0,0,1)$	$(0,0,1)$

Thus, the variables used for the magnetic structure analysis in this work are defined as follows: ***x***_Γ3_ = (*m*^(Γ3,Nd)^_1_, *m*^(Γ3,Ir)^_1_), ***x***_Γ5_ = (*m*^(Γ5,Nd)^_1_, *m*^(Γ5,Nd)^_2_, *m*^(Γ5,Ir)^_1_, *m*^(Γ5,Ir)^_2_), ***x***_Γ7_ = (*m*^(Γ7,Nd)^_1_, *m*^(Γ7,Nd)^_2_, *m*^(Γ7,Nd)^_3_, *m*^(Γ7,Ir)^_1_, *m*^(Γ7,Ir)^_2_, *m*^(Γ7,Ir)^_3_) and ***x***_Γ9_ = (*m*^(Γ9,Nd)^_1_, *m*^(Γ9,Nd)^_2_, *m*^(Γ9,Nd)^_3_, *m*^(Γ9,Ir)^_1_, *m*^(Γ9,Ir)^_2_, *m*^(Γ9,Ir)^_3_). The goal is to determine the globally optimal solutions ***x*** for representations Γ_3_, Γ_5_, Γ_7_ and Γ_9_ using the *I*_mag,obs_ magnitudes listed in Table 1. After that, the solution characterized by the best fit can be selected.

The output values produced by the SDP solver are as follows.

1. The convergence procedure results in the following coefficients:
  $$\begin{array}{c}{{X}_{{\rm{\Gamma }}3}}^{({\rm{opt}})}\approx {{{\boldsymbol{x}}}_{{\rm{\Gamma }}3}}^{({\rm{opt}}1)}{{{\boldsymbol{x}}}_{{\rm{\Gamma }}3}}^{({\rm{opt}}1)T};\\ {{X}_{{\rm{\Gamma }}5}}^{({\rm{opt}})}\approx ({{{\boldsymbol{x}}}_{{\rm{\Gamma }}5}}^{({\rm{opt}}1)}{{x}_{{\rm{\Gamma }}5}}^{({\rm{opt}}1)T}+{{{\boldsymbol{x}}}_{{\rm{\Gamma }}5}}^{({\rm{opt}}2)}{{{\boldsymbol{x}}}_{{\rm{\Gamma }}5}}^{({\rm{opt}}2)T})/2;\\ {{X}_{{\rm{\Gamma }}7}}^{({\rm{opt}})}\approx 0;\\ {{X}_{{\rm{\Gamma }}9}}^{({\rm{opt}})}\approx ({{{\boldsymbol{x}}}_{{\rm{\Gamma }}9}}^{({\rm{opt}}1)}{{{\boldsymbol{x}}}_{{\rm{\Gamma }}9}}^{({\rm{opt}}1)T}+{{{\boldsymbol{x}}}_{{\rm{\Gamma }}9}}^{({\rm{opt}}2)}{{{\boldsymbol{x}}}_{{\rm{\Gamma }}9}}^{({\rm{opt}}2)T}+{{{\boldsymbol{x}}}_{{\rm{\Gamma }}9}}^{({\rm{opt}}3)}{{{\boldsymbol{x}}}_{{\rm{\Gamma }}9}}^{({\rm{opt}}3)T})/3;\end{array}$$
  and
  $$\begin{array}{c}{{{\boldsymbol{x}}}_{{\rm{\Gamma }}3}}^{({\rm{opt}})}=(-\,1.20,\,0.19);\\ {{{\boldsymbol{x}}}_{{\rm{\Gamma }}5}}^{({\rm{opt}}1)}=(0.55,\,0.,\,0.48,\,0.),\,{{{\boldsymbol{x}}}_{{\rm{\Gamma }}5}}^{({\rm{opt}}2)}=(0.,\,0.55,\,0.,\,0.48);\\ {{{\boldsymbol{x}}}_{{\rm{\Gamma }}7}}^{(opt)}=(0.,\,0.,\,0.,\,0.,\,0.,\,0.);\\ {{{\boldsymbol{x}}}_{{\rm{\Gamma }}9}}^{({\rm{opt}}1)}=(0.55,\,0.,\,0.,\,-\,0.52,\,0.,\,0.),\,{{{\boldsymbol{x}}}_{{\rm{\Gamma }}9}}^{({\rm{opt}}2)}=(0.,\,0.55,\,0.,\,0.,\,-\,0.52,\,0.),\\ {{{\boldsymbol{x}}}_{{\rm{\Gamma }}9}}^{({\rm{opt}}3)}=(0.,\,0.,\,0.55,\,0.,\,0.,\,-\,0.52);\end{array}$$
  where the values of ***x*** are expressed the *μ*_B_ units, and the negative sign indicates the nearest ferromagnetic Nd–Ir correlation. While a single optimum solution is determined for representation Γ_3_, multiple solutions are obtained for Γ_5_ and Γ_9_, respectively, in accordance with the rank values of *X*_Γ5_^(opt)^ and *X*_Γ9_^(opt)^, which indicate the existence of an infinite number of solutions with the same objective functions ${{\boldsymbol{x}}}_{{\rm{\Gamma }}5}^{({\rm{opt}})}=\,\cos \,\theta {{\boldsymbol{x}}}_{{\rm{\Gamma }}5}^{({\rm{opt}}1)}+\,\sin \,\theta {{\boldsymbol{x}}}_{{\rm{\Gamma }}5}^{({\rm{opt}}2)}$ and ${{\boldsymbol{x}}}_{{\rm{\Gamma }}9}^{({\rm{opt}})}=\,\cos \,\theta {{\boldsymbol{x}}}_{{\rm{\Gamma }}9}^{({\rm{opt}}1)}+\,\sin \,\theta \,\cos \,\varphi {{\boldsymbol{x}}}_{{\rm{\Gamma }}9}^{({\rm{opt}}2)}+\,\sin \,\theta \,\sin \,\varphi {{\boldsymbol{x}}}_{{\rm{\Gamma }}9}^{({\rm{opt}}3)}$, where $\theta$ and $\varphi$ are arbitrary. Without SDR, it is difficult to find the optimum solutions for this type of problems and prove that better solutions do not exist. Furthermore, the zero magnitude of ***x***_Γ7_^(opt)^ is obtained for representation Γ_7_. Indeed, when ***x***_Γ7_ values are finite (non-zero), the ${I}_{{\rm{mag}},{\rm{cal}}}$ values for ${I}_{{\rm{mag}},{\rm{obs}}}=0$ indices increase more rapidly than those for ${I}_{{\rm{mag}},{\rm{obs}}}\ne 0$ indices. Thus, the SDR method automatically overcomes these issues.
2. The obtained indicator values for representations Γ_3_, Γ_5_, Γ_7_ and Γ_9_ are as follows.

- The corresponding duality gaps determined by the SDP solver are equal to ${\rm{\Delta }}f=2.1\times {10}^{-7}$, $1.4\times {10}^{-6}$, $3.1\times {10}^{-6}$ and $8.0\times {10}^{-7}$, respectively. Their magnitudes are close to zero, indicating that the convergence to the global optimums is achieved.
- The minimized objective function ${f}^{({\rm{gl}})}\underline{\underline{{\rm{def}}}}\,\sum _{k=1}^{N}\,|{I}_{{\rm{mag}},{\rm{obs}}}({Q}_{k})-{I}_{{\rm{mag}},{\rm{cal}}}({Q}_{k})|/{\rm{Err}}[{I}_{{\rm{mag}},{\rm{obs}}}({Q}_{k})]$ = 4.5, 19, 32 and 27, and the more familiar *R*-factor, ${R}_{{\rm{Bragg}}}\underline{\underline{{\rm{def}}}}\,(\sum _{k=1}^{N}|{I}_{\mathrm{mag},\mathrm{obs}}({Q}_{k})-{I}_{\mathrm{mag},\mathrm{cal}}({Q}_{k})|)/(\sum _{k=1}^{N}\,{I}_{\mathrm{mag},\mathrm{obs}}({Q}_{k}))$ = 0.13, 0.63, 1.0 and 0.81, respectively. These results show that the smallest values are obtained for representation Γ_3_.
- The goodness of the fit ${\chi }^{2}\underline{\underline{{\rm{def}}}}\,(\sum _{k=1}^{N}{|{I}_{{\rm{mag}},{\rm{obs}}}({Q}_{k})-{I}_{{\rm{mag}},{\rm{cal}}}({Q}_{k})|}^{2}\,/\,\,{\rm{Err}}{[{I}_{{\rm{mag}},{\rm{obs}}}({Q}_{k})]}^{2})/(N-{n}_{{\rm{\Gamma }}})$ = 2.6, 14, 25 and 20, and the related statistical parameter ${f}^{({\rm{gl}})}/(N-{n}_{{\rm{\Gamma }}})=0.75$, 4.8, 16 and 14, respectively. The value closest to the expected value (1 for ${\chi }^{2}$; $\sqrt{2/\pi }\approx 0.798$ for the latter parameter) is obtained for Γ_3_.

**Figure 3:**
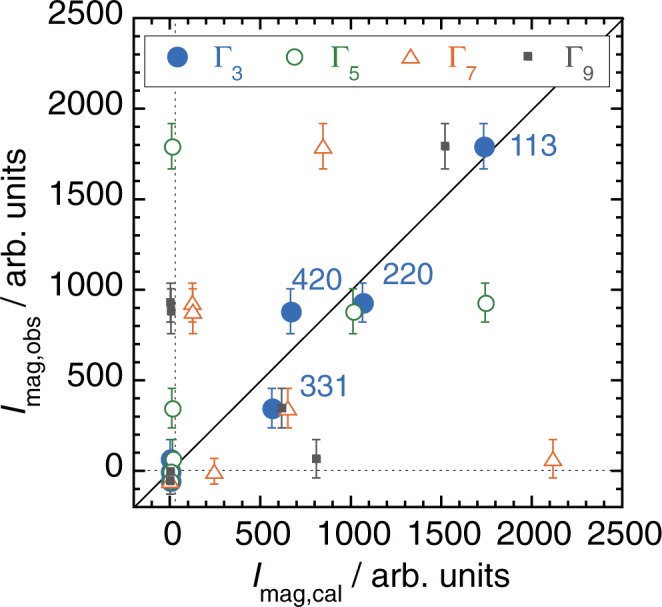
Comparison of the *I*_mag,obs_ (1.4 K) and *I*_mag,cal_ values. The diagonal straight line represents the condition *I*_mag,obs_ = *I*_mag,cal_.

All these facts suggest that Γ_3_ represents the best solution, whereas representations Γ_5_, Γ_7_ and Γ_9_ are excluded for the first time. For clarity, the values of *I*_mag,cal_ are listed in Table 2 and also shown in Fig. 3. The magnitude of *I*_mag,cal-Γ3_ matches *I*_mag,obs_ very well, whereas the globally optimal *I*_mag,cal-Γ5_, *I*_mag,cal-Γ7_ and *I*_mag,cal-Γ9_ parameters substantially differ from the corresponding *I*_mag,obs_ values.

After proving mathematically the uniqueness of representation Γ_3_, the refined values *m*_Nd_ = −1.22(5)*μ*_B_ and *m*_Ir_ = 0.14(5)*μ*_B_ are obtained using the ***x***_Γ3_^(opt)^ values as the initial parameters in the normal least-squares method. These results are comparable with the reported values of *m*_Nd_ = −1.27(1)*μ*_B_ and *m*_Ir_ = 0.34(1)*μ*_B_^34^.

## Discussion

Representation Γ_9_ (the only IR containing ferromagnetic basis vectors^35^) is rejected as the optimal solution despite the existence of weak ferromagnetism^29^. The observed inconsistency suggests that this ferromagnetism is symmetrically decoupled from the bulk magnetic structure (for example, as a surface or interface-protected property). In fact, by performing careful measurements of the macroscopic magnetization and electrical resistivity of isomorphic Cd_2_Os_2_O_7_, it was found that the surface ferromagnetism coupled with novel spin-polarized conductivity emerged on the walls between the all-in all-out and all-out all-in antiferromagnetic domains^36^. The macroscopic observation is consistent with the findings of this study verified both microscopically and mathematically, indicating their potential applicability in domain wall spin electronics.

We discuss the expected application scope of the SDR method. After determining the list of (*h*_mag_, *k*_mag_, *l*_mag_, *I*_mag,obs_) values, the described SDR method can be used to find the corresponding magnetic structures as the global solutions. First, the configurations with ***k***_mag_ = (0, 0, 0) are considered, indicating their high potential applicability in various fields (including magnet materials). Second, this method is not restricted to polarized neutron diffraction experiments, but can be also used in studies involving unpolarized neutrons when the magnetic structure is characterized by ***k***_mag_ ≠ (0, 0, 0) or ***k***_mag_ = (0, 0, 0) with detectable magnetic moment. Both the magnetic and crystallographic structures can be simultaneously refined by the normal least-squares method, in which the obtained SDR solutions are utilized as the initial values of the magnetic structural parameters. Third, the SDR technique is able to easily process thousands of independent variables. Therefore, magnetic structures of arbitrary types can be theoretically determined using the advanced diffractometers that provide a relatively large number of reflection points (even for the target materials with complex compositions).

The diffraction intensities are not represented by quadratic functions of the atomic positions ***r***_*j*_; hence, the SDR technique seems to be inapplicable for determining the values of ***r***_*j*_ (crystal structure). However, the neutron and X-ray diffraction intensities are represented by those of the nuclear and electron densities (generalized crystal structure), respectively. Likewise, a magnetic structure is also generalized to the magnetic moment density. Thus, the SDR technique is expected to enable analysing the global solutions of the generalized structures together with the aforementioned high-volume processing ability, such as protonic/ionic distributions in the conductors and electronic spin-orbital distributions.

## Materials and Methods

### Calculations

The system of quadratic equations was solved using the SDP solver SDPA^37^. To find the global optima of SDP problems, interior point methods were efficiently used^38^.

### Experiments

The polarized neutron elastic scattering experiments were performed using the HB1 thermal neutron three-axis spectrometer located at the High Flux Isotope Reactor (HFIR) of the Oak Ridge National Laboratory (ORNL). Heusler alloy 111 reflection crystals were utilized as the monochromator and analyser. The flipping ratio *R* = 10 was obtained using the nuclear 222 reflection at a paramagnetic temperature of 40 K corresponding to the beam polarization *P* = 0.82. The polarization vector was set parallel to the scattering vector (***P***//***Q***). The incident energy of neutrons was *E*_i_ = 13.5 meV. The horizontal collimator sequence was 48′(open)–80′–80′–240′. A pyrolytic graphite Bragg-reflection filter was used to efficiently eliminate the contamination caused by higher-order wavelengths.

A powder Nd_2_Ir_2_O_7_ sample was synthesized by a solid-state reaction method inside a quartz tube. About 4.8 g of the sample was wrapped in thin aluminium foil and shaped to a hollow cylinder with a thickness of 0.8 mm and diameter of 20 mm to mitigate the effect of the strong neutron absorption of Ir nuclei. The cylinder was stored in an aluminium container filled with He gas and placed under the cold heads of a He-closed-cycle (Displex) or liquid-He-type (Orange) cryostat.

## Electronic supplementary material

Supplementary Information
