# Supplementary material for: A new mathematical approach to finding global solutions of the magnetic structure determination problem
Source: Sci Rep. 2018 Nov 1;8:16228. doi: 10.1038/s41598-018-34443-2 (PMC6212504; doi:10.1038/s41598-018-34443-2)
Supplement: Supplementary file 1 — Supplementary Information [file 41598_2018_34443_MOESM1_ESM.pdf]

Supplementary Information.
A new mathematical approach to finding global solutions of the magnetic structure determination problem

K. Tomiyasu[1], R. Oishi-Tomiyasu[2,3], M. Matsuda[4] & K. Matsuhira[5]

[1]Department of Physics, Tohoku University, Aoba, Sendai 980-8578, Japan.
[2]Faculty of Science, Yamagata University, Yamagata, 990-8560, Japan.
[3]JST, PRESTO, Saitama, 332-0012, Japan.
[4]Neutron Scattering Division, Oak Ridge National Laboratory, Oak Ridge, TN 37831, USA.
[5]Faculty of Engineering, Kyusyu Institute of Technology, Kitakyusyu 804-8550, Japan.

Correspondence and requests for materials should be addressed to K.T. or R.O.-T.
(email: tomiyasu@tohoku.ac.jp or ryoko_tomiyasu@sci.kj.yamagata-u.ac.jp)

I. Details of Mathematical Formulation

A. General descriptions of the SDP and SDR techniques

The following material is based on the known theorems of the field of convex optimization[24]. A typical SDP optimization problem can be represented by one of the following forms:

(i)

$$\begin{cases} \text{Minimize } f_{(\mathrm{i})} \stackrel{\mathrm{def}}{=} C \cdot X \\ \text{subject to: } A_k \cdot X = b_k \quad (k = 1, \dots, N), \\ X \geq 0. \end{cases} \tag{A1}$$

(ii)

$$\begin{cases} \text{Maximize } f_{(\mathrm{ii})} \stackrel{\mathrm{def}}{=} \sum_{k=1}^{N} b_k y_k \\ \text{subject to: } Y + \sum_{k=1}^{N} y_k A_k = C, \\ Y \geq 0. \end{cases} \tag{A2}$$

where $A_k, C, X,$ and Y are the symmetric matrices, and b_k are the real numbers. The inequality $X \geq 0$ indicates that the symmetric matrix X is a positive semidefinite one. The symbol ・ denotes the inner product of the symmetric matrices $X = (X_{ij})$ and $Y = (Y_{ij})$:

$$X \cdot Y \stackrel{\mathrm{def}}{=} Trace(XY) = \sum_{i=1}^{n} \sum_{j=1}^{n} X_{ij} Y_{ij}. \tag{A3}$$

When the parameters A_k, C, and b_k are common for forms (i) and (ii), either (i) or (ii) is called a *primal problem*, while the other one is called a *dual problem*. In this study, problem (A1) is considered the primal problem for clarity. According to the duality theorem, it is possible to determine whether the obtained solutions $X = X^{(\mathrm{opt})}$ and $Y = Y^{(\mathrm{opt})}$ are truly the global optimums by checking if the duality gap $\Delta f = f_{(\mathrm{i})}{}^{(\mathrm{opt})} - f_{(\mathrm{ii})}{}^{(\mathrm{opt})}$ between the optimum values $f_{(\mathrm{i})}{}^{(\mathrm{opt})}$ and $f_{(\mathrm{ii})}{}^{(\mathrm{opt})}$ is close to zero, or more precisely, as small as the square root of the machine epsilon. The weak and strong duality theorems assert that $f_{(\mathrm{i})}{}^{(\mathrm{opt})} \geq f_{(\mathrm{ii})}{}^{(\mathrm{opt})}$ and that if the solutions $X > 0$ and $Y > 0$ satisfying all constraints in forms (i) and (ii), respectively, exist, the SDP problems (A1) and (A2) have equal optimum values. In this study, the existence of such strictly feasible solutions can be easily verified. Further, the complementary slackness theorem assures that

$X^{(opt)}Y^{(opt)} = Y^{(opt)}X^{(opt)} = 0$; hence, the solutions $X^{(opt)}$ and $Y^{(opt)}$ share the same system of eigenvectors. The methodology of interior point methods also ensures that the numerically obtained $X^{(opt)}$ and $Y^{(opt)}$ parameters satisfy the non-degeneracy condition rank $X^{(opt)}$ + rank $Y^{(opt)} = n$.

The SDR technique extends the global-convergence property of SDP to nonconvex optimization problems including the described case; in a broad sense, SDR is a method for approximating the global optimum value. For example, the quadratic programming problem (3) can be written in the following form:

$$\begin{cases} \text{Minimize} \sum_{k=1}^{N} \dfrac{\eta_k^+ + \eta_k^-}{2} \\ \text{subject to:} S_k \cdot X + \dfrac{\mathrm{Err}[I(Q_k)]}{2}(\eta_k^+ - \eta_k^-) = I(Q_k) \quad (1 \le k \le N), \\ X_{ii} + y_i = {R_i}^2, y_i \ge 0 \quad (1 \le i \le n), \\ \eta_k^+ \ge 0, \eta_k^- \ge 0 \quad (1 \le k \le N), \\ X \ge 0, X \text{ is rank 1.} \end{cases} \quad \textbf{(A4)}$$

where S_k and X are the symmetric matrices, and b_k are the real numbers. The last two conditions imply the existence of the vector $\boldsymbol{x} = (x_1, \ldots, x_n)^T$ with $X = \boldsymbol{x}\boldsymbol{x}^T$. If X is the global optimum of problem (A4), the parameters $x_1, \ldots, x_n$ represent the global solution of problem (3), and vice versa. If the rank-1 condition is removed, problem (A4) becomes an SDP one.

The word *relaxation* generally indicates the techniques utilized to obtain information about the global optimums by removing some constraints in order to reduce the degree of computational complexity. More narrowly, *semidefinite relaxation* (SDR) denotes the techniques transforming the nonconvex optimization problem to an SDP one (for example, by removing the rank-1 constraints). After relaxation, the global optimum can be easily determined by the SDP solver. The global optimum value $f^{(gl)}$ is never greater than that of the original problem (A4), because the permitted range of X is simply increased by the relaxation. Thus, the information about the original problem (3) can be obtained using the relaxation of problem (A4). However, the global optimum denoted as $X^{(opt)}$ is sometimes of rank > 1 after relaxation. Furthermore, if multiple global optimums of rank-1 satisfying problem (A4) exist, all the rank-1 solutions are superimposed in $X^{(opt)}$ as follows:

$$\begin{aligned} &X^{(opt)} = X_1 + \cdots + X_s + Z_1 + \cdots + Z_s, \\ &X_i\text{: rank-1 global optimum for problem (A4)} \\ &Z_i\text{: rank} > 1 \text{ global optimum after the relaxation of problem (A4),} \\ &\quad \text{which cannot be represented as a sum of other global optimums of smaller ranks} \end{aligned}$$

The superposition principle described above is based on the two following properties:

- (Linearity of SDP): if both solutions $(X_1, y_k, \eta_k^+, \eta_k^-)$ and $(X_2, z_k, \mu_k^+, \mu_k^-)$ are optimal ones, any combination of $(\lambda_1 X_1 + \lambda_2 X_2, \lambda_1 y_k + \lambda_2 z_k, \lambda_1 \eta_k^+ + \lambda_2 \mu_k^+, \lambda_1 \eta_k^- + \lambda_2 \mu_k^-)$ that satisfies the conditions $\lambda_1 + \lambda_2 = 1$, $\lambda_1 > 0$, and $\lambda_2 > 0$ is also optimal.
- (Strict complementary slackness property): the numerical solution $X^{(opt)}$ and dual optimum $Y^{(opt)}$ obtained by the SDP solver satisfy the condition rank $X^{(opt)}$ + rank $Y^{(opt)} = n$, owing to the methodology of interior point techniques.

The complementary slackness theorem assures that any global optimum solution $\tilde{X}^{(opt)}$ obtained using problem (A4) satisfies the condition, $\tilde{X}^{(opt)}Y^{(opt)} = Y^{(opt)}\tilde{X}^{(opt)} = 0$. The rank-1 $\tilde{X}^{(opt)} = \tilde{x}\tilde{x}^T$ is the global optimum of problem (A4) if and only if the vector $\tilde{x}$ satisfies $Y^{(opt)}\tilde{X}^{(opt)} = 0$ together with the equality constraints specified in problem (A4).

In the case of problem (A4), the optimum value $f^{(gl)}$ cannot be significantly reduced by relaxation since all $\eta_k^+ + \eta_k^-$ values are constrained to be non-negative. Therefore, if the utilized structural model is an appropriate one, the optimum value $f^{(gl)}/(N - n_\Gamma)$ can be as small as $\sqrt{2/\pi}$. SDR can prove the uniqueness of the obtained solution if the corresponding $X^{(opt)}$ is of rank 1; otherwise, the obtained result represents a set of algebraic equations that define all global optimums of problem (A4). Hence, the multiple global optimums can be computed by solving these equations.

B. Derivation of form (3) from form (2)

The following step-by-step transformations explain the derivation:

$$\begin{cases} \text{Minimize} \sum_{k=1}^{N} \dfrac{|I(Q_k) - \boldsymbol{x}^T S \boldsymbol{x}|}{\text{Err}[I(Q_k)]}, \\ \text{subject to: } |x_i| \leq R_i \quad (1 \leq i \leq n). \end{cases}$$

$$\underset{\text{equivalent}}{\Longleftrightarrow} \begin{cases} \text{Minimize} \sum_{k=1}^{N} \varepsilon_k \\ \text{subject to: } \dfrac{I(Q_k) - \boldsymbol{x}^T S_k \boldsymbol{x}}{\text{Err}[I(Q_k)]} \leq \varepsilon_k \quad (1 \leq k \leq N), \\ \dfrac{I(Q_k) - \boldsymbol{x}^T S_k \boldsymbol{x}}{\text{Err}[I(Q_k)]} \geq -\varepsilon_k \quad (1 \leq k \leq N), \\ {x_i}^2 \leq {R_i}^2 \quad (1 \leq i \leq n), \\ \varepsilon_k \geq 0 \quad (1 \leq k \leq N). \end{cases}$$

$$\underset{\text{equivalent}}{\Longleftrightarrow} \begin{cases} \text{Minimize} \sum_{k=1}^{N} \varepsilon_k \\ \text{subject to: } \dfrac{I(Q_k) - \boldsymbol{x}^T S_k \boldsymbol{x}}{\text{Err}[I(Q_k)]} + \eta_k^+ = \varepsilon_k \quad (1 \leq k \leq N), \\ \dfrac{I(Q_k) - \boldsymbol{x}^T S_k \boldsymbol{x}}{\text{Err}[I(Q_k)]} = -\varepsilon_k + \eta_k^- \quad (1 \leq k \leq N), \\ {x_i}^2 + y_i = {R_i}^2, y_i \geq 0 \quad (1 \leq i \leq n), \\ \eta_k^+ \geq 0, \eta_k^- \geq 0, \varepsilon_k \geq 0 \quad (1 \leq k \leq N). \end{cases}$$

Problem (3) is obtained by eliminating the parameter $\varepsilon_k = (\eta_k^+ + \eta_k^-)/2$.

C. The standard SDP form of the relaxation problem (A4)

After taking the relaxation process into account, the following SDP form can be derived from problem (A4):

$$\begin{cases} \text{Minimize} \sum_{k=1}^{N} \dfrac{\eta_k^+ + \eta_k^-}{2} \\ \text{subject to: } S_k \cdot X + \dfrac{\text{Err}[I(Q_k)]}{2} (\eta_k^+ - \eta_k^-) = I(Q_k) \quad (1 \leq k \leq N), \\ X_{ii} + y_i = {R_i}^2, y_i \geq 0 \quad (1 \leq i \leq n), \\ \eta_k^+ \geq 0, \eta_k^- \geq 0 \quad (1 \leq k \leq N), \\ X \geq 0. \end{cases}$$

Below is the standard form (problem (A1)) obtained for the SDP problem described above:

$$
\begin{cases}
\text{Minimize } F_0 \cdot \tilde{X} \\
\text{subject to: } F_k \cdot \tilde{X} = I(Q_k) \quad (1 \le k \le N), \\
F_{k+l} \cdot \tilde{X} = {R_i}^2 \quad (1 \le i \le n), \\
\tilde{X} \ge 0,
\end{cases}
$$

where $\tilde{X}$, F_0, F_k $(1 \le k \le N), F_{M+i}$ $(1 \le i \le n)$ are the following symmetric matrices with two n-by-n and one $2N$-by-$2N$ diagonal blocks. Here, $Z_{n,l}$ is the n-by-n matrix whose (l, l) fields are equal to 1, and the others are filled with zeros. O_n is the n-by-n zero matrix.

$$
\tilde{X} := \left(\begin{array}{c|c|c}
X & & \\ \hline
 & \begin{matrix} y_1 & & \\ & \ddots & \\ & & y_n \end{matrix} & \\ \hline
 & & \begin{matrix} \eta_1^- & & & & \\ & \eta_1^+ & & & \\ & & \ddots & & \\ & & & \eta_N^- & \\ & & & & \eta_N^+ \end{matrix}
\end{array}\right),
$$

$$
F_0 := \left(\begin{array}{c|c|c}
O_n & & \\ \hline
 & O_n & \\ \hline
 & & -\frac{1}{2} I_{2N}
\end{array}\right),
$$

$$
F_k := \left(\begin{array}{c|c|c}
S_k & & \\ \hline
 & O_n & \\ \hline
 & & \begin{matrix} 0 & & & & & & & \\ & \ddots & & & & & & \\ & & 0 & & & & & \\ & & & -\frac{\mathrm{Err}[I(Q_k)]}{2} & & & & \\ & & & & \frac{\mathrm{Err}[I(Q_k)]}{2} & & & \\ & & & & & 0 & & \\ & & & & & & \ddots & \\ & & & & & & & 0 \end{matrix}
\end{array}\right),
$$

$$
F_{k+l} := \left(\begin{array}{c|c|c}
Z_{n,l} & & \\ \hline
 & Z_{n,l} & \\ \hline
 & & O_{2N}
\end{array}\right).
$$

D. Evaluation of the obtained solution

If the duality gap Δf is close to zero, the global convergence of the SDP method has been achieved. Hence, the following conclusions can be made:

- If the globally optimal value of f is not small, the corresponding type of magnetic structure is mathematically not valid; and
- If the f value is small enough, the global optimum solution of problem (2) is numerically obtained using the relations $X^{(\mathrm{opt})} = \boldsymbol{x}\boldsymbol{x}^{\mathrm{T}}$ (if the rank of $X^{(\mathrm{opt})}$ is 1), and $Y^{(\mathrm{opt})}\boldsymbol{x} = 0$ (otherwise). In the former case, the obtained $\boldsymbol{x}$ represents the unique solution. $Y^{(\mathrm{opt})}\boldsymbol{x} = 0$ is a consequence of the complementary slackness theorem.

E. Neutron scattering cross-sections

The neutron scattering cross-sections of nuclear and magnetic reflections can be expressed by the following formula[17]:

$$I_{\mathrm{nuc}}(\boldsymbol{Q}_{\mathrm{nuc}}) = C\,L(\boldsymbol{Q}_{\mathrm{nuc}}) \sum_{\boldsymbol{Q}_{\mathrm{nuc}}} \left| \sum_{j} f_j \exp\left(2\pi i \boldsymbol{Q}_{\mathrm{nuc}} \cdot \boldsymbol{r}_j\right) \right|^2$$

where $\boldsymbol{Q}_{\mathrm{nuc}}$ denotes the nuclear scattering vector $h_{\mathrm{nuc}}\ k_{\mathrm{nuc}}\ l_{\mathrm{nuc}}$, C is the experimental scale constant identical to that for I_{mag}, $L = 1/(\sin 2\theta \sin\theta)$ is the Lorentz factor, 2θ is the scattering angle, the summation of $\boldsymbol{Q}_{\mathrm{nuc}}$ includes multiplicity, f_j is the scattering length of the nucleus of atom j, and $\boldsymbol{r}_j$ is the atomic position. Further,

$$I_{\mathrm{mag}}(\boldsymbol{Q}_{\mathrm{mag}}) = C\,(\gamma_{\mathrm{N}} r_0)^2\, L(\boldsymbol{Q}_{\mathrm{mag}}) \sum_{\boldsymbol{Q}_{\mathrm{mag}}} \sum_{\alpha} \sum_{\beta} \left(\delta_{\alpha\beta} - \frac{Q_{\mathrm{mag},\alpha} Q_{\mathrm{mag},\beta}}{|\boldsymbol{Q}_{\mathrm{mag}}|^2} \right) \times$$
$$\left\{ \sum_j \left(\frac{m_{j,\alpha}}{2}\right) F_{\mathrm{M},j}(\boldsymbol{Q}_{\mathrm{mag}}) \exp(2\pi i \boldsymbol{Q}_{\mathrm{mag}} \cdot \boldsymbol{r}_j) \right\} \left\{ \sum_j \left(\frac{m_{j,\beta}}{2}\right) F_{\mathrm{M},j}(\boldsymbol{Q}_{\mathrm{mag}}) \exp(-2\pi i \boldsymbol{Q}_{\mathrm{mag}} \cdot \boldsymbol{r}_j) \right\}$$

where $\boldsymbol{Q}_{\mathrm{mag}}$ denotes the magnetic scattering vector $h_{\mathrm{mag}}\ k_{\mathrm{mag}}\ l_{\mathrm{mag}}$, γ_{N} is the gyromagnetic ratio of the neutron, r_0 is the classical electron radius, the summation of $\boldsymbol{Q}_{\mathrm{mag}}$ includes multiplicity, α and β are the Cartesian directions, $\boldsymbol{m}_j$ is the magnetic moment at $\boldsymbol{r}_j$, and $F_{\mathrm{M},j}$ is the magnetic form factor of atom j. Using these formulas and the standard least-squares method, it is possible to numerically determine the common C value and other structural parameters from I_{nuc}, while I_{mag} produces the coefficient matrix S as a quadratic function of $m_{j,\alpha}$. Thus, magnetic structure analysis can be performed as problem (2).

II. Separation of I_{mag} and I_{nuc} values

In this experiment, the polarization vector $\boldsymbol{P}$ is oriented parallel to the scattering vector $\boldsymbol{Q}$ to eliminate the complex nuclear-magnetic interference term from the neutron scattering cross-section[17]. As a result, the following equations are obtained,

$$I_{\mathrm{OFF}} = (1 - r_{\mathrm{mix}})\, I_{\mathrm{nuc}} + r_{\mathrm{mix}}\, I_{\mathrm{mag}}$$
$$I_{\mathrm{ON}} = r_{\mathrm{mix}}\, I_{\mathrm{nuc}} + (1 - r_{\mathrm{mix}})\, I_{\mathrm{mag}}.$$

Hence,

$$I_{\mathrm{nuc}} = \{(1 - r_{\mathrm{mix}})\, I_{\mathrm{OFF}} - r_{\mathrm{mix}}\, I_{\mathrm{mag}}\}/\{2(1 - r_{\mathrm{mix}}) - 1\}$$
$$I_{\mathrm{mag}} = \{-r_{\mathrm{mix}}\, I_{\mathrm{OFF}} + (1 - r_{\mathrm{mix}})\, I_{\mathrm{ON}}\}\, /\{2(1 - r_{\mathrm{mix}}) - 1\},$$

where $R = N_+/N_-$ is the flipping ratio, $P = (N_+ - N_-)/(N_+ + N_-)$ is the polarization value, and $r_{\mathrm{mix}} = N_-/(N_+ + N_-)$ is the mixing rate of minority-spin neutrons in a beam. The value of r_{mix} is obtained at a paramagnetic temperature of 40 K ($I_{\mathrm{mag}} = 0$). The additional neutron scattering data are shown in Fig. S1, and the corresponding magnetic and nuclear intensities are listed in Tables S1 and S2, respectively.

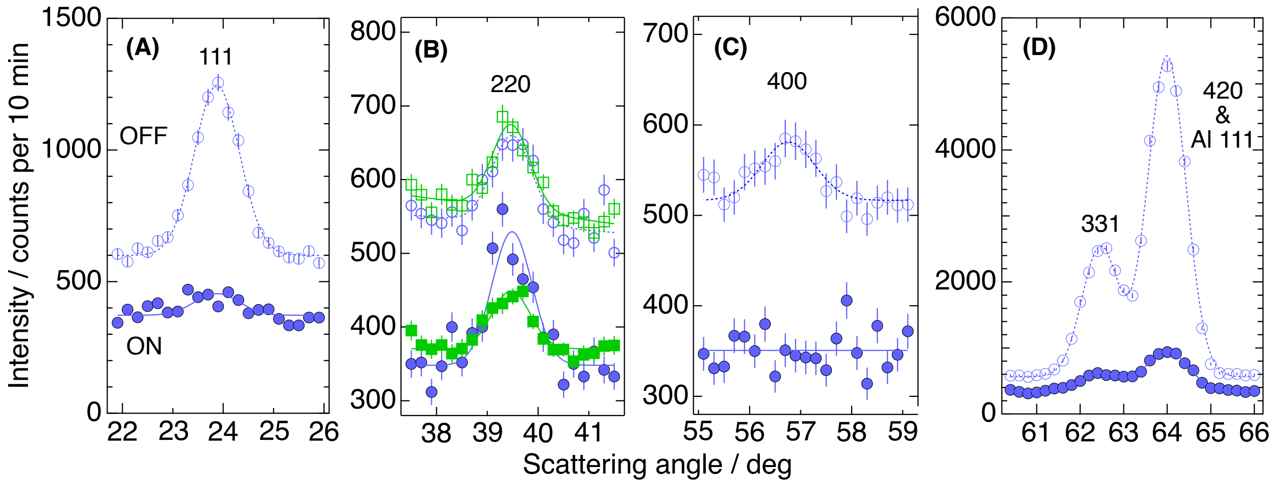

Figure S1. Additional neutron scattering data. The open and solid purple circles denote the I_{OFF} and I_{ON} channels measured at a temperature of 1.4 K, respectively. The green squares shown in panel (B) represent the 6-K data. The I_{OFF} values presented in panels (A), (B), (C), and (D) are vertically shifted by 200, 150, 150, and 200 counts, respectively, for clarity.

Table S1. Experimentally observed and calculated integrated magnetic reflection intensities. The $I_{mag,cal}$ values are obtained for the final refinement values in the Γ_3 representation; m_{Nd} = 1.22(5)μ_B and m_{Ir} = 0.14(5)μ_B at T = 1.4 K; m_{Nd} = 0.80(5)μ_B and m_{Ir} = 0.19(5)μ_B at T = 6 K.

h_{mag} k_{mag} l_{mag}	$I_{mag,obs}$ (1.4 K)	$I_{mag,cal}$ (1.4 K)
111	0	0
200	0	0
220	932	1062
113	1796	1732
222	0	0
400	0	0
331	349	562
420	884	665
**h_{mag} k_{mag} l_{mag}**	**$I_{mag,obs}$ (6 K)**	**$I_{mag,cal}$ (6 K)**
220	347	347
113	912	912
222	0	0

Table S2. Experimentally observed and calculated integrated nuclear reflection intensities. The crystallographic atomic positions are provided for Nd 16*d* (1/2,1/2,1/2), Ir 16*c* (0,0,0), six seventh of oxygens O 48*f* (*x*,1/8,1/8), and one seventh of oxygen O' 8*b* (3/8,3/8,3/8) in the space group $Fd\bar{3}m$ (No. 227). The $I_{nuc,cal}$ values are obtained for a = 10.36(1) Å and x = 0.333(1) at T = 1.4 K and for a = 10.36(1) Å and x = 0.334(4) at T = 6 K.

h_{nuc} k_{nuc} l_{nuc}	$I_{nuc,obs}$ (1.4 K)	$I_{nuc,cal}$ (1.4 K)
111	3996	4802
200	0	0
220	553	709
113	10292	11193
222	62920	62809
400	470	95
331	11581	10936
**h_{nuc} k_{nuc} l_{nuc}**	**$I_{nuc,obs}$ (6 K)**	**$I_{nuc,cal}$ (6 K)**
220	564	777
113	10545	11172
222	62922	62808

III. Addendum to magnetic structure analysis

The described SDR method was also used to confirm that representation Γ_3 remained the global solution in the expanded cases of $\Gamma_3 + \Gamma_5$, $\Gamma_3 + \Gamma_7$, and $\Gamma_3 + \Gamma_9$.

The magnitude of m_{Ir} is relatively low. However, as shown in refs.[33,34], its value strongly affects the diffraction intensity because it contains the Nd-Ir interference term. For simplicity, let us consider the case of $I_{mag} \propto (f_{mag,Nd} \pm f_{mag,Ir})^2$, where $f_{mag,Nd}$ and $f_{mag,Ir}$ are linearly dependent on m_{Nd} and m_{Ir}, respectively. As a result, the expression $I_{mag} \propto (f_{mag,Nd})^2 \pm 2(f_{mag,Nd} f_{mag,Ir}) + (f_{mag,Ir})^2 \cong f_{mag,Nd}{}^2 \pm 2(f_{mag,Nd} f_{mag,Ir})$ is obtained. Thus, the intensity change is proportional to the term $(f_{mag,Ir})^1$ and not $(f_{mag,Ir})^2$, owing to the relatively large ordered magnetic moment of Nd.

The number of reflections detected at a temperature of 6 K is very small. However, because the system remains in the same physical phase between 1.4 K and 6 K, it is possible to determine the 6-K magnetic structure with the same Γ_3 symmetry represented by only two basis vectors. According to the obtained results, the value of m_{Nd} significantly increases with this small temperature decrease, whereas the magnitude of m_{Ir} remains unchanged. The observed temperature dependence supports the results of previous neutron scattering and muon spin resonance studies, in which the Ir moment primarily produces a magnetic order at a temperature below T_{MI} = 36 K, accompanied by the formation of the Nd magnetic structure below $T \approx 15$ K through Nd–Ir ferromagnetic interactions[33,S1].

References

S1. Asih, R., Adam, N., Mohd-Tajudin, S. S., Sari, D. P., Matsuhira, K., Guo, H., Wakeshima, M., Hinatsu, Y., Nakano, T., Nozue, Y., Sulaiman, S., Mohamed-Ibrahim, M. I., Biswas, P. K. & Watanabe, I. Magnetic Moments and Ordered States in Pyrochlore Iridates $Nd_2Ir_2O_7$ and $Sm_2Ir_2O_7$ Studied by Muon-Spin Relaxation. *J. Phys. Soc. Jpn.* **86**, 024705 (2017).
